# Gendered Perspectives on Men's Changing Familial Roles in Postwar England, *c*.1950–1990

**DOI:** 10.1111/1468-0424.12333

**Published:** 2018-03-13

**Authors:** Angela Davis, Laura King

## Introduction

This article examines the ways in which men and women remember men's place in, and experiences of, family life in postwar England during a period when a new ideal of fatherhood arguably emerged. Based on forty‐four oral history testimonies with men and women, this article adds a new dimension to existing literature in gender history by closely examining men's *and* women's perspectives on the same issues. Focusing on decisions around family planning, experiences of pregnancy and birth, and the division of labour in the home, the article analyses how men and women understood their respective roles as parents‐to‐be and as new parents; how they negotiated the expectations of those around them; and the extent to which the gendering of childcare responsibilities persisted in the decades between 1950 and 1990.

Despite the advent of second‐wave feminism and multiple challenges to stereotyped roles for men and women within the home over this period, it is clear from this material that parenting often remained a gendered and gendering experience for individuals. While a considerable change in men and women's perceptions of an idealised role for fathers appears to have occurred in the decades after the Second World War, this perception of increased paternal involvement was not always matched by an increase in fathers’ actual involvement. The involvement of the father in parenting continued to vary considerably at the individual level, as had been the case in the first half of the century. Interviewees arguably used and incorporated changing gendered norms, and created gendered and non‐gendered identities to manage a workable private life as they became parents. The identity of ‘parent’ could allow for a flexible sharing of duties in childcare, but most interviewees presented themselves as ‘mother’ or ‘father’ within the context of a retrospective interview.

The article contributes to growing historiography on the role of fathers within modern British family life.^1^ Historians such as Joanne Begiato (Bailey), Julie‐Marie Strange, Lynn Abrams, Margaret Williamson and Laura King have demonstrated that affectionate fathers can be found in numerous periods and throughout Britain. Irrespective of a family's social and economic circumstances, close bonds could be formed between fathers and children.^2^ Of course, this was never true of all families, and alongside such affectionate fathers were men who were distant and even abusive.^3^ Simultaneously, fatherhood fluctuated in its prominence within popular culture, as fathers were demonised and celebrated at different points in time.^4^ Elizabeth Roberts and, more recently, Laura King locate the beginnings of the modern shift in men's involvement in family life and a new emphasis on shared decision‐making between parents in the interwar period. In postwar Britain, there was a more intensified interest in fatherhood, particularly amongst the media, and a ‘family‐orientated’ masculinity emerged.^5^ Fatherhood was a convenient way to help men position themselves and be positioned socially within ‘normal’, peacetime, family life, and away from soldierhood and war – in Britain and beyond. Arguably this increased cultural emphasis on men's family roles and identities echoed an increasing involvement of men in family life, but there remained substantial diversity over the degree to which men played an active role in the home in the 1940s and 1950s.^6^ Research from the 1960s found that while men were taking on an important share in childcare, this rarely represented an equal division of labour.^7^

Further, pioneering sociological studies focusing specifically on fatherhood in the 1970s and 1980s presented a mixed picture.^8^ Outlining the impetus for his study, Brian Jackson noted, ‘Doubtless every generation has to discover fatherhood afresh; but there seemed reasonable grounds for suspecting that some significant shift might just be afoot’.^9^ In contrast, Charlie Lewis was more sceptical about claims that the 1970s and 1980s had seen a sudden and novel increase in men's involvement in family life, commenting that such claims were ‘as old and perhaps as prominent as the notion of patriarchy’, and that women remained primarily responsible for the home and children.^10^ This article takes a historical perspective to interrogate gendered perspectives on change and continuity to men's roles from the late 1940s to the 1980s in relation to three main areas: family planning and conception; pregnancy and birth; and infant care.

By the end of the twentieth century, the nuclear family itself was destabilised: Jane Lewis, for example, writing in 2003, argued that ‘artificial reproduction and the increasingly messy nature of intimate relationships mean that the family is no longer “natural”; biological and social motherhood and fatherhood can be separated’.^11^ Lewis noted that most children spent at least some time growing up in a ‘non‐traditional’ household.^12^ However, the reasons why these changes occurred, the point in time in which change began, and the degree of continuity which has remained, are all issues of intense debate.^13^ In recent years scholars have turned their attention to the postwar period, using gender as a means to analyse developments in sex and marriage, family, and the relationship between men and women at home and in the workplace.^14^ Despite this interest, there has not been the same attention paid to the relationship between men, work and home that there has been for women. If we are to fully understand these social changes, considering how men's place in the home was conceived is crucial.^15^

This article therefore extends existing scholarship on postwar British society and the gender relationships which underpinned it by focusing on the role of men within family life. The article interrogates the spaces between ideals and lived experience, evident in the oral history material, and offers a new perspective on debates around continuity and change in men's domestic role. It argues that, while ideals of fatherhood and the role of men in the home dramatically shifted in the postwar period, there were nonetheless important continuities in the gendered division of labour within the home. This period saw a new ideal of highly involved and ‘hands on’ fatherhood in England, and this sense of change was highlighted by interviewees. However, overriding this semblance of change were two factors. Firstly, though ideals and practices of both motherhood and fatherhood were changing, there was little evidence of these roles merging or the gendering of parenthood practices and identities disappearing. Secondly, the division of labour and responsibility for children in practice changed little: women continued to do the majority of childcare in this period. Factors such as class and region influenced expectations, but there was also significant variation between families of the same background, suggesting that interpersonal dynamics and individual behaviour also played a role.

## Methodology and sources

This exploration of men's changing familial roles is based on an analysis of two oral history collections, one with men and one with women, which together comprise a total of forty‐four testimonies. The two authors conducted these oral history interviews independently, and have subsequently brought them together for further analysis for this co‐authored article. Twenty‐one of the interviews were with fathers and twenty‐three with mothers. The interviews with men were conducted in 2013–2014 specifically by Laura King for a study about men's experiences of their partners’ pregnancies, childbirth and infant care. In three cases, participants’ partners also took part in the interview, and in a further case, one participant's wife was present in the room, and contributed at one point. Male interviewees were found through local community groups, online advertising and advertising in community spaces such as libraries, as well as snowballing. The sample were mostly located in the north of England, but had lived throughout the country and abroad in small numbers too. Many were highly educated and a significant number were or had been engaged with socialist and feminist politics. Most couples in the sample brought their children up together in at least the early years of their lives, though a significant minority subsequently separated. Indeed, given that the subject matter of the original research project focused on men and family life, it can be reasonably assumed that this group of men were especially concerned with their roles as fathers.

The interviews with women were carried out between 2002 and 2009 for a project about motherhood in post‐1945 England by Angela Davis. The interviews with women were all one‐on‐one with the exception of Eve whose husband was also present. The female interviewees were found through community and women's groups and snowballing with interviewees putting other women they knew into contact with the interviewer. Interviewees were selected to include a range of locations and educational backgrounds. However, educated middle‐class women most often volunteered themselves to contribute to the research and most were living in the south of England at the time of the interview. Snowballing further encouraged the over‐representation of the middle class and highly educated, as women tended to recommend women from the same socioeconomic background as themselves. None of the women entered into motherhood as single parents and the majority (although not all) were married to the fathers of their children at the time of their births. A significant number had been single parents at some point due to death or divorce, and some women had later entered into new relationships. The majority of interviewees for both projects were white and most originated from the United Kingdom. These two sets of interviews were subsequently re‐used to provide a comparative perspective specifically for this co‐authored article.^16^ Both authors examined all interviews in order to provide a new analysis on this subject matter; adding to the complexity and richness of the research and demonstrating the potential for collaborative work across separate oral history research projects.

All interviewees became parents between 1948 and 1990. Most interviewees and their children were born in England.^17^ They were drawn from various occupational backgrounds, which would largely be classified as middle class, and the sample was, overall, a highly educated one. Middle‐class couples are of particular interest here because the narrative of change in gender roles that sociologists believed they were documenting in postwar Britain was presented as being most advanced for these groups.^18^ In their 1973 book *The Symmetrical Family*, Michael Young and Peter Willmott suggested that the division of labour between men and women was becoming less marked and that the professional middle class was the vanguard of this change. They argued that men and women were moving towards more or less equal roles, both working in the home and outside it, with the home becoming central to social life and social identities. This trend would, therefore, have been most visible amongst middle‐class couples, such as the men and women interviewed here, should it have occurred.

In oral history, variables such as social background, geographic location, ethnicity and religion intersect with gender as men and women tell their life stories. Class is identified as an important but ‘fuzzy’ variable, with a large degree of overlap between different groups.^19^ This was particularly so in the latter half of the twentieth century, as cultural norms relating to parenting held by different class groups were to some extent converging after the Second World War, although differences in practices remained.^20^ Like class identities, it is clear that local and regional variations remained important, and it is easy to find evidence that individual experiences were shaped by the local context in which they lived. In this article, we take into account such differences, although we predominantly draw on gender as our key question of analysis to focus on the difference in men and women's perspectives.

The interviews were conducted in two periods, which, despite being chronologically close, differed markedly in social, economic and political terms. This shaped the interviewees’ reflections. The interviews with women by Angela Davis were undertaken in a generally more optimistic time where new initiatives in childcare and support for working families were encouraging women to enter the workforce and the jobs were available for them to do so. The Labour government of 1997–2010 prioritised support for working parents generally, and working mothers in particular, in the form of childcare and tax credits. In his Comprehensive Spending Review speech on 12 July 2004, the Chancellor of the Exchequer, Gordon Brown, declared the twenty‐first century as the era of universal childcare.^21^ The women interviewed at this time were therefore living in a period of change which facilitated a new relationship between home and work for many women. The increased support for working mothers that they saw in comparison to when they were raising their own children may have encouraged them to focus on the increased opportunities they perceived as being open to women. As Jane Lewis has argued, the ideal of a male breadwinner, and mother as primary caregiver, dominated attitudes about the family for much of the twentieth century. In both samples, in the 1940s and 1950s, only an exceptional few of the mothers and the wives/partners of the men interviewed worked when their children were below school age. The majority took a period of time out of paid labour before returning, usually part‐time, once their children were at school. This reflected national trends and social expectations; formal marriage bars were no longer in place, but social norms encouraged women to leave work upon marriage or, increasingly as the period progressed, upon having children. Sample tables from the 1951 census showed that 4.5 per cent of the women with children under one year of age were in paid work, and less than a quarter of married women in total. As the period progressed more mothers were in paid work. By the end of the 1970s one in six women returned to employment within six months of having their first baby, rising to one in four at twelve months. Between 1988 and 1998, the employment rate for women with children under five rose from 36 per cent to 50 per cent. Thus, the state support for working women in the early 2000s arguably contributed to a sense of optimism about gender equality in the workplace and home.

In contrast the men were interviewed by Laura King in the aftermath of the 2007–8 financial crisis, a period when many of the services set up to support working families by the previous administration were at risk under the Conservative‐led Coalition. Working mothers were arguably worst affected by the crisis and resulting tax rises, cuts to benefits and public services, and rising unemployment. Indeed female unemployment rose from 678,000 in 2008 to 1.08 million in 2013, a level previously seen in 1988.^22^ This challenged contemporary assumptions about the inevitability of women's labour market participation. However, this was also a period in which the Coalition government was combining traditional Conservative values promoting the private family unit, and Liberal Democrat initiatives to promote gender equality.^23^ Fathers’ roles were being renegotiated, not least through the offer of extended parental leave to be shared with their partners. Few men interviewed, however, acknowledged or seemed aware of these policy initiatives.

While there are significant differences in the collections, the interviews are in other ways comparable. Both sets of interviews were semi‐structured and followed the model described by Penny Summerfield.^24^ Focusing on the life cycle, the interview format encouraged the respondents to consider their own lives in comparison to their parents and children, and within a wider trajectory of social change. All interviews centred on the interviewee's experience of parenting, placing their subjectivity as mother or father at the forefront, and asked interviewees to place their subjective experiences in relation to their partner. Our analysis acknowledges the individuality of each collection, and each interview, and pays attention to the fact that oral history interviews are co‐created between interviewee and interviewer and tell as much about the moment they were undertaken as past events. Nonetheless, we believe oral history is a particularly effective methodology to access subjective attitudes and experiences. The benefits of being able to compare the accounts of men and women outweigh the limitations brought by the fact the interviews were not conducted at the same time or by the same interviewer. Our analysis demonstrates the possibilities of scholarly collaboration to realise the potential of existing data sets as new research questions arise, particularly as there are strong ethical arguments for the reuse of interviews to maximise the opportunity this data presents. Both collections offer rich resources and considered together offer an opportunity to explore men's and women's perspectives of men's changing role in the family alongside one another.

Concentrating on three themes – family planning and conception; pregnancy and childbirth; and infant care – this article examines how men's role in the home and family life was understood by each generation, from the perspectives of both men and women. Despite social changes in the decades after the Second World War, from the impact of second‐wave feminism to the rise of cohabitation, there was also striking continuity in parenting practices and the different roles performed by men and women into the 1980s. The article suggests this was based on particular beliefs about inherent gendered differences. Yet, despite the discussion of such gender‐based differences, both men and women insisted at the time of interview that there had been substantial and significant changes in men's roles over their lifetimes. Indeed, as King has argued, change can occur in ideas and practices of masculinity and femininity without any substantial shift in how the two are positioned in relation to each other.^25^ In other words, there was change in understandings of motherhood and fatherhood, but the relationship between these roles remained similar. Across the accounts of both men and women, there was an almost Whiggish sense of continual improvement in the behaviour and attitudes of fathers. This article critically examines such notions of change; compares men's and women's articulation of change and continuity; and argues that both the men and women interviewed highlighted differences in their respective roles in family life, even if they also spoke of equality and a substantial shift from previous generations. Men were likely to defer to their wives or partners whom they viewed as ultimately responsible for and more expert in looking after children and home. Women expected their husbands or partners to contribute, but branded this as ‘help’. Thus, although we can find evidence of changing patterns of behaviour, this change was limited by a specifically gendered understanding of an inherent and continuing difference in the way men and women contributed to and were responsible for family life.

## Family planning and conception

Decisions to start a family and discussions around what that family will look like are an obvious starting point to understand the ways in which gender roles and expectations influence parenting dynamics. The respective roles of men and women in controlling fertility in the twentieth century have been much debated.^26^ Kate Fisher has suggested that from the First World War to the 1960s men were often seen as responsible for securing contraception and largely taking care of this aspect of marital relations.^27^ Furthermore, Fisher suggests that family planning was often tacitly negotiated between wife and husband, rather than being part of an open discussion.^28^ In a separate study, Fisher and Simon Szreter found there were marked class and regional differences in attitudes towards contraception and the actual methods used.^29^ Based on their interviews with men and women born between 1901 and 1931, undertaken for their study of sexual behaviour for the period 1918 to 1963, Fisher and Szreter found that, even among this older generation, ‘middle‐class women were more prepared to be directly involved in contraception and as a result family limitation was initially approached as a joint problem for couples in discussion with each other’.^30^

Fisher and Szreter were interviewing an earlier generation than the mainly middle‐class couples discussed in this article, who were born between 1924 and 1952. A majority claimed that family size was a shared decision, and many of the men and women interviewed spoke of relatively open discussions about sexuality and family planning with their partner. This arguably reflects the middle‐class composition of the interviewees in this study, but potentially indicates significant change in attitudes towards fertility and sexuality in the postwar period. Notably, men and women alike talked at length about wanting a family and what that family might look like; and there was little evidence of men's ambivalence to having children that Marcus Collins found in an earlier period.^31^ While the testimonies are present selves remembering what they thought and how they acted several decades earlier, and are not a record of their actual feelings or behaviour at this time, these interviewees indicated that they now think, and want to present themselves as having thought then, that family size should be a shared decision.

Marjorie, born in 1931, had the first of her four children in Oxford in 1959. Her husband was working at the University where she had also studied. She said the family was planned around her husband's career, but that they were both involved in the decision making: ‘to start with we knew we couldn't possibly cope with having children because my husband was doing … his third year of his doctorate’. When he finished they thought ‘it didn't matter so desperately if a baby came along’. Women who had children later in the period presented a similar picture of shared decision making. Karen, born in 1945, had four children, born in 1967, 1970, 1984 and 1985. When asked whether she and her husband had planned to have two and then two later she said, ‘we had actually talked about it, right at the beginning … It was something that we thought would be nice’.

Male interviewees also used this language of shared decision‐making regarding family planning. Born in 1942, in a working‐class area of Leeds, Mike had worked his way up through the clothing industry. Mike discussed his and his wife's approach to family planning in the early 1970s and placed this in a wider generational narrative of change. He tried continually to involve his wife, who was also present, in the conversation, and said ‘we thought things out very, very carefully, didn't we?’, adding, ‘I don't think my mum and dad did, so much’. Mike and his wife agreed she should give up work when the children were young, demonstrating the persistence of more traditional gender roles within this framework of shared decision making. Edward, born 1940, and his wife Lily, whose only child was born in 1976, also presented their family planning as a joint endeavour when asked whether they had discussed having children, saying ‘we weren't going to have one for six months after we got married’. Problems with fertility meant their son was actually conceived some years later. The fact they were interviewed together likely encouraged this retelling of a shared experience: throughout their account of having a family, they spoke in agreement, filling in the narrative together. However, Edward also suggested that medical staff treated him as uninvolved when they were having fertility problems, indicating continuing norms could be in tension with their relationship. Born in 1950, Alex met his wife in the 1970s and they married in 1975, after she had divorced her first husband. Alex used a shared language of ‘we’ in describing the couple's decisions: ‘We had no desperate desire to have children … but we thought actually if you don't, you've got long, long twilight years to regret it’. He did not differentiate between his and his wife's desires, presenting a united decision‐making process throughout the interview. They had four children born between 1979 and 1986.

The decision over family size was also presented as one that was reached jointly. Hayley was born in 1926 and brought up in Welwyn Garden City, Hertfordshire. Having worked as a research chemist in Bristol, she left work when the family moved to Essex and had their first child in 1956. Hayley recalled their decision to have three children: ‘I wanted to have four really, coz I was one of four, and he wasn't that keen because he was one of two [laughing] so we compromised with three’. Diana's three children were born in 1958, 1959 and 1964. The first two were born when the couple were living in South Oxfordshire to fit with her husband's career in the RAF. Their last child was born in Yemen where he was then stationed. When asked if they planned their children as they did, Diana answered, ‘yes we did, in fact we decided you know we'd have one, and then we'd have another one and then said yes, we'll have another one [laughing] … we were going to have four. But we ended up with a dog for the youngest one … Coz I think I said no’. For middle‐class couples, such as Hayley and Diana, starting their families in the late 1950s, the decision to have children and the number of children they would have was assumed to be something which couples would have control over, and it would be a decision they would reach together.

However within this overall picture of joint decision‐making, some women, such as Diana in her recollection that she ‘said no’, also hinted that they had ultimate responsibility for family planning. There were also some men who indicated that their wives’ held greater responsibility in decisions about having children. Fred had one daughter in 1961, and although he wanted more, and in particular a boy, his wife wanted to return to her career in nursing. He added, ‘she had given me one child, as it were, and that was my lot! I didn't argue with her’. This trend became more pronounced as the period progressed. For example, when asked whether she and her husband had planned to have their first child in 1960, a year after marrying, Claire replied, ‘Oh yes, we'd planned to have children as soon as we got married’. In total the couple had six children. Discussing whether this was also planned she said, ‘Well we meant to have eight, but then we had six and decided enough was enough’. She added, jokingly, that ‘We were bad at birth control [laughing]. I didn't want to go on the pill because I saw my female friends getting grumpy and putting on weight’. While presenting her choice not to go on the pill in a humorous way, Claire also implied that whilst they envisioned their future family together, the form of contraception was her choice. Pam had two children born in 1977 and 1983. When discussing family size she focused on her own wishes, and did not discuss her husband's views: ‘I definitely didn't want one so I suppose yes I did want two, and I wanted to have them close together because I didn't want a big gap like I had [with my sibling]’. However later in the interview Pam recalled speaking to her doctor and she referred to the decision on starting a family as one that ‘we’ (she and her husband) made, indicating that while contraception may have increasingly been viewed as a woman's responsibility, this was still understood within a context of joint decision‐making. She explained: ‘the doctor said to me don't you think you've left it a bit late, because I was thirty [laughs] … I said, “No I don't think so”. We wanted to wait so we had a few years together first and we had a house and we were ready for a baby’. Pam's comments indicate that, while she had her own views on the subject which she expected to realise, she also thought that starting a family was something which couples should decide upon together.

Interviewees’ often implicit recognition of their power over such decisions perhaps reflected changing contraceptive technologies, and particularly the invention of the contraceptive pill. This was first available in Britain in 1961, although only for married women, and could enable women to take more control over their own fertilty. By 1964 an estimated 480,000 women were taking the drug.^32^ While the pill was not the first contraceptive method under a woman's control, and while there is evidence that the educated, middle‐class women were already aware of methods of family limitation, Hera Cook believes the introduction of the contraceptive pill was a turning point because of its increased efficacy and the opportunity women had to make autonomous decisions about their own fertility with ease.^33^ Camilla, who had three children in the early 1960s, discussed the impact of the pill: ‘I mean after the pill came out, it was my saving, things of course changed and I didn't have any more, but I really don't know [laughs] what would have happened otherwise’.^34^ It is noteworthy that she spoke in the first‐person singular here, and described the pill as changing her life, with no mention of her husband. The women's liberation movement also encouraged women to take back control over their bodies in the context of their relationships and encounters with medical practitioners, which may have affected women's actions at the time and the way those women spoke retrospectively.^35^ Donald also indicated that it was his partner who had the ultimate say in family size. The couple met through a socialist group and it was partly the excitement of being part of this socialist, feminist movement that made him reluctant to change this life by having children. Yet ultimately they had one son in 1983 because, ‘she really wanted to, and she's a very strong woman, erm, so I did’. Donald implied that while a joint decision, it was largely led by the desires of his partner.

In their narratives of family planning, men's and women's accounts suggest decision‐making was, already by the 1950s and increasingly in the decades after, talked about as shared, particularly for middle‐class couples, even if their accounts intimated that women had the final say. This supports Fisher and Szreter's findings that, while male responsibility for contraception had been the norm before the 1960s (and this was most pronounced in working‐class couples), a gender reversal occurred in subsequent decades, beginning within the educated middle classes. As female contraceptive methods became better known and more widely available, women were increasingly seen as responsible for contraception.^36^ This change, already underway by 1950s with technologies such as the cap, accelerated with the introduction of the contraceptive pill in the 1960s. Interviewees who had children in the 1970s and 1980s indicated contraception was seen as a woman's responsibility with the associated consequence that women were now able to have the final say about family size. Indeed, this also reflected the context of the 2000s and 2010s when the interviews took place, when media reports suggested that young women were also seen to be responsible for male methods of birth control.^37^ However, while in practice the interviewees indicated that by the later decades of the twentieth century the pill gave women a greater ability to control family size, this was not the ideal they presented in their accounts. Both men and women thought having children should be a joint decision and this belief shaped their narratives.

## Pregnancy and birth

Although ‘fathercraft’ classes were established as early as the interwar period, it was not until later in the twentieth century that men were encouraged to take a more active role in pregnancy and birth. This development parallels increasing provision of antenatal care for women.^38^ For women who were pregnant in the 1950s and 1960s antenatal care was often limited to check‐ups and did not include antenatal education or childbirth preparation classes. Men were therefore not excluded from such classes; they did not exist. Fiona went to private antenatal classes in Brighton in the late 1960s because there was nothing similar provided on the National Health Service (NHS). She said her husband ‘thought it was rather funny all these classes’, but did go to ‘one or two’. The birth educator Sheila Kitzinger started the first Natural Childbirth Trust (the National Childbirth Trust after 1961) couples’ antenatal classes in the country, in Oxfordshire, in the late 1950s.^39^ As classes increased in number from the 1960s, both privately and on the NHS, various factors deterred men's involvement. Even if the classes took place in the evening, it was difficult for men who worked long hours or had a long commute to attend. Indeed this was also a problem for working women. In addition, the classes were seen as being for women: the medical aspects of pregnancy were still framed as women's business. Liz had three children born between 1973 and 1980. She said, ‘antenatal classes were just for women. Men weren't encouraged to go’. Edward discussed his role during his wife's pregnancy in the mid‐1970s ‘only as a taxi’. Men were not welcomed at the antenatal classes his wife attended, though he was there for the birth. His wife highlighted that he was teaching at that time, and ‘so going with me to appointments a] wasn't the done thing anyway and b] [he] couldn't, because he couldn't have time off school’. Barry described feeling ‘partially’ involved in his wife's pregnancy in 1972, noting that his work was ‘taking up quite a bit of time’. He thought he ‘did all that I felt was necessary’. He talked to his wife about the pregnancy, but it was not practical for him to attend classes, and ultimately he reflected that he did not want to do so at the time.

Ben, whose first two children were born in Reading, discussed a different kind of experience in the 1970s, borne out of a different kind of relationship dynamic he felt he had with his wife. He felt involved, noting he and his wife always did things together, and went to as many classes as possible. Other men hinted that much as they might have felt involved, their partner remained in charge of everything related to the pregnancy and the baby. This was the case for James, who went to the requisite classes leading up to the birth of his first child in 1986, where he made friends and felt involved, but noted that his wife ‘was organising most stuff, so I was going along and helping out as best as possible’. James's description of his role as ‘helping out’ was not so different from the language men and women used to describe the role of fathers thirty years before, again indicating that despite substantial change in expectations and practices of fathers around pregnancy, there were also continuities.

In the 1950s and 1960s most men were not present at their children's births whether at home and in hospital. In Newson and Newson's 1950s study of Nottingham families, fathers were present in 13 per cent of home births.^40^ Few hospitals let any lay person, male or female, into the delivery at this time. Tania's second, third and fourth children were born at home in 1953, 1954 and 1962. She said, ‘of course, in those days they didn't have the husband there. Although he was in the house when [my second son] was born … but they didn't have them in the room’. Through using the word ‘they’ Tania indicated that it was the choice of the midwife and perhaps wider social convention rather than a personal decision that she and her husband made. Similarly Brian described what happened when his wife gave birth at home in 1967. He noted, ‘I was there when Eve started in labour but her mother came, now her mother was a bit of a matriarch … I saw the head, you know, appear and Eve's mother said you can get out, she said, this is nothing to do with men’.

Home births could, however, open up the possibility of men's presence, and King argues it was here that change first occurred.^41^ Glenda, for example, described her husband's support during the birth of her two children in 1952 and 1954: ‘I valued my husband's presence … because I mean you're not left on your own when you're at home and I could howl and scream and moan whereas nurses always put a stiff upper lip on it, he was always very kind’. Likewise, Harry and Rose described how the young midwife involved in their second child's birth in 1952 was keen to involve Harry. Though reluctant at first, Harry agreed to be there, and concluded that apart from their wedding day, ‘it was the loveliest experience of my life, and I could recommend anybody to attend, I could truthfully now’. Whilst there was some degree of choice for women giving birth at home, those doing so at hospital were usually alone. No relative was allowed to be present. Fiona, for example, described how when her first child was born in 1966, ‘there was no fathers, you couldn't possibly have a father in the room, they'll get in the way or whatever’. Fiona said her husband ‘didn't even know he had a son until nine o'clock that morning when he rang up’.

By the latter part of the 1960s more husbands were present in hospitals for at least part of labour if not the delivery. Amanda's first child was born in hospital in Bristol in 1965. She said, ‘my husband did actually come in, which was probably unusual, it wasn't really planned … he came up to visit me, and they just sort of put a mask on him’. From the 1970s onwards attitudes changed more extensively. Fathers found they were not just allowed but indeed expected to be present at their children's births, reflecting something of a shift in the understanding of their relative importance in family life. By the 1970s and early 1980s, when almost all births took place in hospital, over half of men attended their children's births, and evidence suggests that by the end of the 1990s about 90 per cent did so.^42^

By the 1980s the majority of husbands (and partners) interviewed were present at their children's births. Bev's first child was born in 1987 after a number of years of fertility treatment and the couple were both very involved with the pregnancy. Bev explained, ‘We knew it was a boy … Right from early on. And we'd chosen his name and everything’. However the birth challenged their shared involvement as her husband was pushed into the background and left as a marginal figure:

I went in for my caesarean, things weren't going too well, and they said to my husband, ‘It'll either be [our son] coming out or me coming out. He wouldn't get us both out alive’. And … then when he was … called in … I was in the recovery room. He was called in. Told to sit by my bed … he thought they'd left him there on his own to say his goodbyes … And at that point, he just didn't know our son had been born healthy.

Bev said by the time their second child was born in 1990 many changes in care had taken place. She was now able to make a ‘very detailed birth plan’ which the hospital ‘strictly followed’ and her husband was present throughout. Bev said she was told her husband could have even been present during a caesarean and added ‘You know, even within those few years. Things had changed greatly’.

Men's attendance at birth was an area in which new expectations of fathers were realised in changing practices. The 1970s saw a dramatic and rapid shift towards men's presence becoming the norm, from a small minority attending pre‐1970 to over 70 per cent of men attending at least some part of the birth in numerous studies from the 1970s onwards.^43^ The increase in men's attendance at classes, appointments and birth itself indicates a new belief in the role of husbands as emotional and practical support for their wives, and furthermore, in their rights and responsibilities as fathers. Being equally involved in decision‐making around family size, and moreover, playing some role in the pregnancy and birth could indicate their emotional commitment to family life. Yet, men's accounts of childbirth indicate they felt there remained a limit to their involvement. Whilst women highlighted men were getting involved, many fathers felt rather on the sidelines during this time; George, for example, described his feeling of being ‘a bit like a spare thumb’ and ‘helpless’ during his second child's birth in 1975. He added ‘I felt I should be more involved than I was … I just felt like I should be able to do rather more than I was able to do’. This was a common sentiment; James even joked that he was probably a ‘hindrance’ when his children were born in the late 1980s. The experience of birth could be an emotional and positive experience for men, but could also underline their relative lack of involvement bringing to the forefront a tension between an ideal of an emotionally engaged and active father and a more complex reality. This tension became starker in the retrospective interview because pressures towards actively engaged fathers, in birth and beyond, had increased from the 1990s onwards.

## Caring for babies and children

Numerous researchers have examined men's involvement in infant care, from various disciplinary perspectives.^44^ This involvement should not be conflated with domestic labour; many men were happier to look after children than take on housework such as cleaning.^45^ Immediately after the birth, the absence of formal paternity leave schemes meant men found it difficult to take time off work to be with their wives and new babies, even if they wanted to do so. Though many men worked in the days and weeks following the arrival of their baby, others did find ways to spend time with their families, taking holiday or other forms of leave. By 1983, one study found only thirteen of 230 employed fathers took no time off, with most using holiday or unpaid leave.^46^ Some employers, such as British Rail and the Greater London Council, offered private paternity schemes by the early 1980s.^47^ Indeed, one interviewee, Reg, whose first child was born in 1979, was involved in campaigning for paternity leave for teachers as a member of the National Union of Teachers. He was astounded at opposition on the grounds that men might have more children in order to get time off. Such a move would formalise the practice in which head teachers ‘would give you a nod’ and men could have a few days off.

Reflecting this position nationally, many interviewees recalled that fathers had limited and in some cases no time off after the births of their children, particularly in the first part of this period. Penny's first child was born in 1957 in Cambridge, when her husband had just started a new job 100 miles away. While she thought her husband took to the care of their new baby more easily than she did, the distance and his lack of time off meant she was primarily responsible for the child's care; ‘he used to come at the weekends and lend a hand’. Ben also had children in the 1970s and though he and his wife took a very shared approach to childcare, this was limited by the demands of his employer and wider societal expectations. He recalled ‘as far as most employers was concerned in those days, I'd done my job nine months earlier’, as ‘they didn't consider I had any responsibility after that’. Interviewees recalled that it was assumed men would only be needed at home when subsequent children were born so they could look after the older siblings when their wives were in hospital.

The belief men were less able to care for babies (as opposed to older children) was linked to widely held assumptions in mid‐century Britain that women were the ‘natural’ carers of infants. Female interviewees did not lay great stress on biological difference in their accounts, possibly because they internalised this view and thought it was so taken for granted they did not need to discuss it, or because they attributed the division of labour to other causes (see below). However, some male interviewees reflected more explicitly on how apparently ‘natural’, biological differences could affect their approach to parenting, in comparison to their wives or partners. Men often described themselves as feeling less naturally able to look after a baby compared to their wives. Barry, for example, described how when he visited his wife and baby in hospital (in 1972), it was ‘a bit too soon’ to pick him up. He described how his mother‐in‐law stayed with them, and overall he was ‘quite happy to leave it to the ladies’. Nick and Cath, interviewed together, agreed that she had been in charge when their children were young in the 1970s. As Nick described, ‘she knew what she was doing! I didn't know’, and highlighted how she taught him to parent. Greater expertise was often given as the reason for women's greater involvement in childcare. For Mike, whose two children were born in the 1970s, the gendered differences between him and his wife were a positive; he described how ‘we both contributed different things. I thought like a bloke, she thought like a woman’. Simon spoke in quite a similar way, describing his wife as ‘a lot, lot more maternal, Mother Earth kind of thing’. He liked taking his three children, born 1987, 1989 and 1990, to the park and other activities, tapping into an older tradition of fatherhood as linked to entertainment and public outings.^48^

As well as highlighting the greater expertise or ability of their wives and partners in infant care, employment often cropped up unprompted when men discussed the division of labour. When asked about the birth of his daughter in 1961, Fred described what happened, and then moved on to discuss his job and how busy he was at this time, noting ‘you felt guilty if you took time off to do private things, even the birth of a baby’. Henry described, ‘From the start I wanted to be involved, but having said that I worked [outside the home] inordinately hard, so [my wife] carried it … they owe much more to her than to me’. Henry had been able to be substantially involved with the practical childcare of his daughter, born when he was a student. Yet, overall, he summarised the limits on his involvement throughout most of their family life with deference to his wife's expertise in parenting when he said, ‘I was the first reserve really, rather than the partner. A sort of stand in’. Hilda also reflected how her husband's role as family breadwinner determined how involved he could be at home. She said her children (born in 1967 and 1970) ‘didn't get to see dad an awful lot because well they were in bed when he went to work and they were in bed when he came home from work … and I can remember thinking god I might as well be a single parent’. Hilda's comments reveal how taken for granted the male breadwinner was for many couples at this time, as her reference to single parenthood did not acknowledge that lone parents often also had to work outside the home to support their families. Her testimony is particularly interesting as Hilda herself had been raised by a working mother who had been widowed. Her account may be more indicative of the climate in which she was interviewed, the early 2000s, where more was expected of men in the home, than contemporary experience and expectations in the late 1960s and 1970s.

The division of labour early in the life of a new family, coupled with men's potential separation from pregnancy and childbirth, could set a gendered pattern for subsequent family life. For many, women's role still remained primarily in the home, and this reinforced the distinction of men's involvement as ‘help’.^49^ This negotiation of women's position was often tied to discussions about their paid work. Alf discussed how ‘of course’ his wife had stopped paid work when she was pregnant with their first child in 1961, as they both felt she should not work outside the home. They took pride in the fact that neither of their children needed their own key until well into their teenage years, and agreed this was a sign of being ‘good parents’. Like many female interviewees, Mary reflected on Alf's involvement as ‘help’. Whilst Alf wondered, explicitly noting this was fifty years ago, ‘probably I didn't do as much nappy changing as maybe I ought to have done’, Mary reflected that ‘you didn't do as much nappy changing as what they do today’, but ‘if I asked you to do something you would do it for me’. This comment reflects a perception of substantial change in childcare responsibilities between the time Alf and Mary had small children, and the time of interview. Overall they agreed he was a ‘hands on father’ for the time, and the only limit on this was time spent earning money for the family. Donald and his partner, both self‐identified feminists, who shared housework, noted that in caring for their son, born in 1983, ‘we certainly didn't share equally’, and simply highlighted that ‘she wanted to be with him’. Interestingly, he said that he discussed those early days of childcare with his partner in preparation for the interview, as he was a bit ‘hazy’ in his recollection of that period, perhaps also reflecting his belief that his partner was ultimately responsible for, or perhaps more invested in family life.

Women also talked of being responsible for the home and family. Fiona, who had three children between 1966 and 1970, said although her husband

…was quite happy to give the boys their breakfast and that sort of thing, so I could have a bit of a lie‐in in the morning … and then he'd help put the kids to bed and that sort of thing, but that was the absolute limit … But I think that was normal. That was what you expected.

Some men and women had a strict understanding about their respective roles with women being responsible for the home and men engaged in paid work outside it. Amanda stopped work after her first child was born in the mid‐1960s because she and her then husband had thought she should be at home to care for the family, a decision she had agreed with at the time, but which in her interview she said she now regretted.

Whilst many couples performed very distinct roles in family life, a minority sought to create a different approach. Their desire to more equally balance parenting duties arguably reflected the challenge presented by the feminist movement. Though as Jeffrey Weeks argues, ‘The rise of the Women's Liberation Movement, from its founding conference at Ruskin College, Oxford in 1969, was undoubtedly one of the most important political and cultural events of the 1960s and 1970s, and indeed of the late twentieth century as a whole’, its influence did not translate into changes in lived experience for many couples and only a minority of interviewees, mostly those who actively identified with feminism, created a more shared, equal approach to the division of labour.^50^ When Yvonne and her husband were raising their three children in the 1960s he took an equal part in childcare which enabled her to return to university. Yvonne said, he was ‘very domesticated’. Further examples of this sharing of breadwinning and homemaking came predominately from couples who had had children in the 1970s and 1980s. Kelly, a university lecturer in Manchester, said she managed to combine work and motherhood in the 1970s with the help of her husband before and after they separated. However, men and women were not treated equally in the labour market, even at the end of the century. Kelly recalled she was discriminated against throughout her career, earning less than her male colleagues doing the same job. This is despite the fact that as a professional woman, working full‐time, Kelly was in a privileged position. While on the one hand equal opportunity policies and structural changes in the economy have generally favoured women, on the other hand the pursuit of labour market deregulation has had an adverse effect, and the increasing employment opportunities for women at the end of the twentieth century were experienced very differently for women of different social economic backgrounds.^51^ Engagement with feminist ideas and experience of higher education could enable couples to create a shared and equal approach to parenting, but the wider take up of such ideas also remained limited and continuing structural and societal barriers made this difficult.

Moreover, even at the end of the period, men and women who described taking a more equal approach to managing childcare with paid work implied that they were doing something unusual. Bev was the main breadwinner after her two children were born in 1987 and 1990. She said, ‘it was unusual for a lady to go back to work. And certainly to go back full‐time’. Indeed for some couples, sharing childcare was part of a wider desire to live differently to their peers and reject contemporary social conventions, reflecting the counter‐culture ideals of the period but also indicative of the bias within our sample. Daniel took this further, and prioritised the raising and home‐schooling of his children and stepchildren in the 1980s over paid work. Their ‘very alternative lifestyle’ was often at odds with friends and neighbours but allowed him to spend extensive time with their children. However, Daniel also reflected that he was the ‘junior partner’ because his wife already had two children when they met. He also reflected he focused on being indulgent and fun, and thought looking back that, ‘I should be sharing some of the worry so that she could be sharing some of the fun, but … it didn't play out that way’. Malcolm lived in a commune with his partner when his first child was born in 1975, and they tried to create a different style of living, influenced by feminist and left‐wing thought which challenged traditional family structures. He and his partner swapped part‐time and full‐time working roles between them, and shared care of their children. However, on reflection, Malcolm felt that he brought something different to parenting than his partner through his masculinity, such as his reliability, strength and being calm‐headed in a crisis. He had realised that his own father's somewhat ‘old‐fashioned’ masculine ideals were something positive he could use himself.

When discussing their or their husband's involvement, both male and female interviewees often placed this within a narrative of generational change and the belief fathers were doing more childcare as the century progressed. Asked if her husband had done more in the home when they were raising their children, who were born in the 1950s, than her father, Glenda answered:

Well two things really, my father's generation wouldn't expect to, but also my husband was very, very, my father wasn't bad, but [my husband] was a very kind, pleasant, easy‐going man, I was very lucky. And it didn't ever occur to us not to share things. But I think the better ones of today's dads … they do more than my husband did.

It is noteworthy that even within her account of change Glenda made clear that she was not talking about all men: she implies that there were men less involved than her own father, that her husband was unusual in sharing childcare with her and that even today it is the ‘better’ fathers that have a more involved role. Henry, who had four children in the 1960s and 1970s, similarly discussed the pattern of change in his own family, describing how ‘I wasn't like my father in that I did nothing, but I didn't do much. My own children do much more than I did’. Across the different periods of two sets of interviews could be found a narrative of progress in terms of men's childcare responsibilities. However within these accounts of change there was awareness that men's and women's patterns and hours of work in and outside the home remained different. For example Lynne, who had one child in 1973, reflected:

… my son actually only has a four‐day week and he does an amazing amount with the children you know relatively amazing. It's the kind of thing that still there's a lot of gender prejudice I think, I'm using the word amazing if he was their mother I wouldn't be saying it presumably, you know what I mean?’

The acknowledgement that women were still seen as primarily responsible for the home and children, even in the twenty‐first century when the interviews were conducted, is evidence of the continuity in parental practices that the oral testimonies revealed. Only a minority of couples, fewer than five out of forty‐four, really created a shared approach to parenting. Most others felt their differentiated but mutually supportive roles were the most effective approach for them. It also highlights the ambiguity of the meanings of home for women as a place whereby they could exert their authority, but one that could also confine them.

Such contradictions have long existed. Lynn Abrams noted, in respect to women in the early twentieth century, that, ‘it was within these interconnected spheres [of family and community] that women, paradoxically, possessed greatest autonomy. The domestic sphere was a context of both subordination and the springboard for ideas about liberation’.^52^ In her analysis of working‐class Lancashire marriages in the first half of the century Roberts highlighted, ‘the woman exerted significant power, not so much from legal rights as from moral force … There was, for example, no law which stated a working‐class woman should control her family's finances, and yet in every family but one in the sample this was the case’.^53^ Contrasting the decades before and after 1940, Roberts argued that rising living standards of the postwar period meant women lost control over the family budget and family life more generally.^54^ However Diana Gittins has queried Roberts’ interpretation, positing that, ‘there is a confusion between power and responsibility and that the fact that such power was contingent on the husband's willingness to hand over his wages’.^55^ Our findings for the postwar period reflect Gittins’ critique that responsibility and power are not synonymous. Being in charge or more expert in family matters did not mean women were necessarily in a position of power, as our female interviewees’ accounts show: both family dynamics and the social and economic structures surrounding families ensured the continuity of a model of the family in which women were expected to be primary caregivers, despite some dramatic changes in family life throughout our period.

## Conclusion

Our interviews indicate that even after 1940, and amongst the middle‐class couples who constitute most of our sample, there remained the idea that women were responsible for matters relating to family life. There was a sense of changing roles, as men were happier getting their hands dirty with nappies, and demonstrated a more hands‐on approach to caring for babies than previous generations. There were also some dramatic shifts, such as men's increased presence during childbirth. In considering their experiences, most interviewees told their stories in a longer personal context of generational change, contrasting their experiences with their grandparents, parents and children. However, these changes were limited by deep continuities in gender roles, with mothers remaining more responsible for childcare despite some increase in men's involvement. Indeed, most men at some point in the interview suggested the interviewer should speak to their partners, given the topic of family and childbirth, despite the focus of the research on men's subjective experiences of these life events. Many couples, throughout this period, welcomed the opportunity for the mother to stay at home to look after small children, and were pleased their financial position allowed this. The circumstances couples found themselves in often led them towards a more ‘traditional’ version of family life than they might have liked, as taking leave from work was often difficult for men, and most interviewees still placed great significance on men's roles as breadwinners. Overall, men's emotional engagement with family life was high – they played an active role in making decisions about having children, and wanted to be involved in pregnancy and childbirth, often as far as their paid work and societal expectations allowed. However, when examining actions rather than intentions, focusing on our third section of infant care, we can see the gendering effect of parenting, as men were most likely to defer to their partners’ expertise and ‘help’ in parenting when they had satisfied their breadwinner role. By bringing together two sets of interview data we can see how deep continuities in gendered ways of thinking about family life remained. Indeed, comparing these two different interview collections alongside one another shows how prevalent and enduring such views were: they were iterated by both sexes, they were expressed by interviewees living in different parts of the country, and were shared by interviewees who were reflecting back from periods that, although chronologically close, were quite different socially and politically. For us as scholars working on the complementary but distinct research areas of fatherhood and motherhood, sharing our data in this way has both revealed the commonalities in experience for men and women living in postwar England, but also encouraged us to be more critical in our analysis of the differences that did occur.

Three aspects of individual accounts are of particular significance in this regard. Firstly, a large proportion of the male interviewees throughout the period, though insistent that they were very involved in domestic labour and childcare when their children were little, noted and accepted that their wives or partners remained ‘in charge’ and responsible for family life. Their attitudes reflected wider societal beliefs that childrearing and childbearing were women's issues and that women were responsible for the home and family, as can be seen in the fact antenatal classes took place in working hours which men could not attend or in the limited paternity leave men received. Secondly, and leading on from this, whilst female interviewees increasingly welcomed their husband or partner's high involvement in housework and childcare, and were displeased by those who did not live up to this, such involvement was consistently branded as ‘help’ and ‘hands on’ men were viewed as unusual or special. Men remained secondary and voluntary in their involvement.^56^ Thirdly, therefore, men and women did not explain the different roles they performed in the family and the continuity of such gender difference (despite changes to these roles) in the same way. Men highlighted a qualitative and quantitative difference between motherhood and fatherhood. They emphasised women's expertise in homemaking and childcare, and deferred to this expertise. They also stressed that their work often limited their involvement. While women also talked about apparently innate gendered difference, they tended to highlight structural reasons, such as lack of affordable childcare or hostile labour market practices, as explaining why their generation of women had been primarily responsible for homemaking and childrearing and conversely why their husbands had been less involved.

These factors, of course, are by no means unique to England. Many social, economic and demographic trends witnessed in postwar England were experienced in other countries and this story of substantial change confined within deep continuities in gender roles is common to other parts of the Western and Anglophone world.^57^ As a recent collection on parenting between generations demonstrated, throughout different cultures, one's own parents’ attitudes and behaviour are the major influence on becoming and being a parent, and so change can be gradual.^58^ As Lewis has argued, while there have been ‘rapid and dramatic’ changes in family structure and the labour market in the last quarter of the twentieth century ‘gender is about more than families, or women's employment, or indeed ‘reconciling’ family and (paid) work’. She continues that thinking about the ‘the gendered division of care work, more often unpaid than paid’ is crucial.^59^ The testimonies of men and women analysed here therefore also demonstrate the continuities in attitudes towards gendered familial roles throughout the twentieth century, and into the twenty‐first. While they reveal tender and caring companionship within marriage and attentive and involved fathers, this remained within a framework where women were seen as ultimately responsible for the day‐to‐day care of children.

## Notes

This paper developed out of the Gender and History workshop on ‘Men at Home’ hosted by Raffaella Sarti at the University of Urbino in 2013. Many thanks to the participants for useful comments on the early research for this article and to the other colleagues, particularly the editorial staff at *Gender & History*, who have helped us in its preparation. We would also like to thank the Wellcome Trust for their support, both through the Wellcome Trust Strategic Award at the Centre for the History of Medicine, University of Warwick which funded Laura King's initial research into this area, and for supporting Angela Davis through a University Award at the University of Warwick, during which the article was written.

## Footnotes

1 While our research was focused on the English experience, we address the wider British context when referring to scholarship that does encompass Britain and not solely England.

2 Lynn Abrams, ‘“There was Nobody like my Daddy”: Fathers, the Family and the Marginalisation of Men in Modern Scotland’, *Scottish Historical Review* 78 (1999), pp. 219–42; Joanne Bailey, ‘“A Very Sensible Man”: Imagining Fatherhood in England *c*.1750–1830’, *History* 95 (2010), pp. 267–92; Laura King, *Family Men. Fatherhood and Masculinity in Britain, 1914–1960* (Oxford: Oxford University Press, 2015); Julie‐Marie Strange, *Fatherhood and the British Working Class, 1865–1914* (Cambridge: Cambridge University Press, 2015); Margaret Williamson, ‘“He was Good with the Bairns”: Fatherhood in an Ironstone Mining Community, 1918–1960’, *North East History* 32 (1998), pp. 87–108.

3 King, *Family Men*, pp. 101–3, 126–7.

4 On the lack of prominence of fatherhood in popular culture in the nineteenth century, see Claudia Nelson, *Invisible Men: Fatherhood in Victorian Periodicals, 1850–1910* (Athens, GA and London: University of Georgia Press, 1995), esp. pp. 51–4. On its increasing prominence in the twentieth century, see Laura King, ‘Hidden Fathers? The Significance of Fatherhood in Mid‐Twentieth‐Century Britain’, *Contemporary British History* 26 (2012), pp. 25–46.

5 King, *Family Men*, p. 5; Laura King, ‘“Now You See a Great Many Men Pushing Their Pram Proudly”: Family‐Orientated Masculinity Represented and Experienced in Mid‐Twentieth‐Century Britain’, *Cultural and Social History* 10 (2013), pp. 599–617.

6 King, *Family Men*, esp. pp. 51–88; King, ‘Now You See’, pp. 605–10.

7 Michael Fogarty, Rhona Rapoport and Robert N. Rapoport, *Sex, Career and Family* (London: Allen & Unwin, 1971).

8 Brian Jackson, *Fatherhood* (London: Allen & Unwin, 1983); Charlie Lewis, *Becoming a Father* (London: Open University Press, 1986); Lorna McKee and Margaret O'Brien (eds), *The Father Figure* (London: Tavistock Publications, 1982).

9 Jackson, *Fatherhood*, p. 7.

10 Lewis, *Becoming a Father*, pp. 5, 98.

11 Jane Lewis, *Should We Worry about Family Change?* (Toronto: University of Toronto Press, 2003), pp. 3–4.

12 Lewis, *Should We Worry*, p. 4.

13 For example, see the debate between Pat Thane and Samantha Callan and Rebecca Probert about the timing of continuity and change. Pat Thane, *Happy Families? History and Family Policy* (London: British Academy, 2010) and Rebecca Probert and Samantha Callan, *History and Family: Setting the Records Straight. A rebuttal to the British Academy Pamphlet* Happy Families? (London: The Centre for Social Justice, 2011).

14 See Stephen Brooke, ‘Gender and Working Class Identity in Britain during the 1950s’, *Journal of Social History* 34 (2001), pp. 773–95; Claire Langhamer, ‘Love, Selfhood and Authenticity in Post‐War Britain’, *Cultural and Social History* 9 (2012), pp. 277–97 and Simon Szreter and Kate Fisher, *Sex Before the Sexual Revolution: Intimate Life in England 1918–63* (Cambridge: Cambridge University Press, 2010).

15 Claire Langhamer, ‘Feelings, Women and Work in the Long 1950s’, *Women's History Review* 26 (2016), pp. 77–92 and; Helen McCarthy, ‘Women, Marriage and Paid Work in Post‐war Britain’, *Women's History Review* 26 (2016), pp. 46–61.

16 Various scholars have considered the benefits and limitations of reusing oral history interviews. While offering rich potential for research, the process also brings challenges in ethics, interpretation and understanding. April Gallwey, ‘The Rewards of Using Archived Oral Histories in Research: The Case of the Millennium Memory Bank’, *Oral History* 41 (2013), pp. 37–50; Joanna Bornat, Parvati Raghuram and Leroi Henry, ‘Revisiting the Archives – Opportunities and Challenges: A Case Study from the History of Geriatric Medicine’, *Sociological Research Online* 17 (2012), pp. 1–12, here p. 11; Ronald J. Grele, ‘On Using Oral History Collections: An Introduction’, *The Journal of American History* 74 (1987), pp. 570–8; Peter Jackson, Graham Smith and Sarah E. Olive, ‘*Families Remembering Food: Reusing Secondary Data’*, Working paper (2007), http://www.sheffield.ac.uk/polopoly_fs/1.145076!/file/FRF-reuse-paper-WP-.pdf; Niamh Moore, ‘(Re)Using Qualitative Data?’, *Sociological Research Online* 12 (2007), http://www.socresonline.org.uk/12/3/1.html.

17 The men and women were born in locations evenly distributed around England (with a small number born outside England). There was a bias among the children for those of the male interviewees to be born in the north of England and those of the female interviewees to be born in the south (with a small number born abroad).

18 See Angela Davis, ‘A Critical Perspective on British Social Surveys and Community Studies and their Accounts of Married Life *c*.1945–1970’, *Cultural and Social History* 6 (2009), pp. 47–64.

19 See Jon Lawrence, ‘Class, “Affluence” and the Study of Everyday Life in Britain, *c*.1930–64’, *Cultural and Social History* 10 (2013), pp. 273–299, here p. 275.

20 Brooke, ‘Gender and Working Class Identity’, p. 773; Davis, ‘A Critical Perspective’, p. 54; Ronald Fletcher, *Britain in the Sixties: The Family and Marriage: An Analysis and Moral Assessment* (Harmondsworth: Penguin Books, 1962), p. 127; John Goldthorpe, David Lockwood, Frank Bechhofer and Jennifer Platt, *The Affluent Worker in the Class Structure* (Cambridge: Cambridge University Press, 1969), p. 163; Geoffrey Gorer, *Exploring English Character* (London: The Cresset Press, 1955), p. 162; Lawrence, ‘Class’, p. 285; Szreter and Fisher, *Sex Before the Sexual Revolution*, p. 29.

21 Peter Moss, ‘A New Era for Universal Childcare? Childcare and Early Years Services’, in *Setting the Scene: A Vision of Universal Children's Spaces* (London: Daycare Trust, 2004), p. 3.

22 Oxfam, *The True Cost of Austerity and Inequality UK Case Study*, September 2013, https://www.oxfam.org/sites/www.oxfam.org/files/cs-true-cost-austerity-inequality-uk-120913-en.pdf, p. 3.

23 Angela Davis, ‘The Party of the Family’, *Open Democracy*, 19 October 2012, https://www.opendemocracy.net/ourkingdom/angela-davis/party-of-family.

24 Penny Summerfield, *Reconstructing Women's Wartime Lives* (Manchester: Manchester University Press, 1998), p. 1–42. The majority of the interviews were one‐on‐one and typically one to two hours long. Four interviews for the fathers’ project were conducted with couples and there was a further interview when a partner was in the room. One interview for the mothers’ project was conducted with a couple. To enable informed consent the aims of the research were explained to potential respondents in advance of the interview. Interviewees were given the chance to specify any restrictions they wished to make on their contributions. A unique identifying pseudonym has been used to preserve the anonymity of the interviewees for both collections. Recordings and transcripts are held by the interviewers in both cases, in line with the conditions placed by the interviewees at the time of the interview. Both samples were self‐selecting in that all men and women had volunteered to be interviewed.

25 King, *Family Men*, pp. 5–6.

26 Hera Cook, *The Long Sexual Revolution: English Women, Sex, and Contraception 1800–1975* (Oxford: Oxford University Press, 2004), esp. ch.7, pp. 165–186; Diana Gittins, *Fair Sex: Family Size and Structure 1900–39* (London: St Martin's Press, 1982); Wally Seccombe, *Weathering the Storm: Working‐Class Families from the Industrial Revolution to the Fertility Decline* (London: Verso, 1993).

27 Kate Fisher, *Birth Control, Sex and Marriage in Britain 1918–1960* (Oxford: Oxford University Press, 2006), pp. 228–30. Also see Kate Fisher, ‘“She Was Quite Satisfied with the Arrangements I Made”: Gender and Birth Control in Britain 1920–50’, *Past and Present* 169 (2000), pp. 161–93.

28 Kate Fisher, ‘Uncertain Aims and Tacit Negotiation: Birth Control Practices in Britain, 1925–1950’, *Population and Development Review* 26 (2000), pp. 295–317.

29 Szreter and Fisher, *Sex before the Sexual Revolution*, pp. 229–267.

30 Szreter and Fisher, *Sex before the Sexual Revolution*, pp. 253–4.

31 Marcus Collins, *Modern Love: An Intimate History of Men and Women in Twentieth‐Century Britain* (London: Atlantic Books, 2003), p. 109.

32 Cook, *Long Sexual Revolution*, pp. 268 and 272.

33 Cook, *Long Sexual Revolution*, pp. 268 and 272.

34 Cook, *Long Sexual Revolution*, pp. 281–325.

35 Existing scholarship in this area has particularly focused on the United States. For example, Sandra Morgen, *Into Our Own Hands: The Women's Health Movement in the United States, 1969–1990* (New Brunswick: Rutgers University Press, 2002) and Jennifer Nelson, *More than Medicine: A History of the Feminist Women's Health Movement* (New York: New York University Press, 2015). On pregnancy testing and the Women's Liberation Movement, see Jesse Olszynko‐Gryn, ‘The Feminist Appropriation of Pregnancy Testing in 1970s Britain’, *Women's History Review* (2017), pp. 1–26.

36 Szreter and Fisher, *Sex before the Sexual Revolution*, p. 267.

37 For example, see http://www.telegraph.co.uk/women/womens-health/11681713/Birth-control-Men-dont-want-to-talk-about-contraception.html

38 Rosaline S. Barbour, ‘Fathers: The Emergence of a New Consumer Group’, in Jo Garcia, Robert Kilpatrick and Martin Richards (eds), *The Politics of Maternity Care* (Oxford: Clarendon Press, 1990), pp. 202–16, here p. 212.

39 Angela Davis, *Modern Motherhood: Women and Family in England c.1945–2000* (Manchester: Manchester University Press, 2012), p. 73.

40 John Newson and Elizabeth Newson, *Patterns of Infant Care* (London: Pelican, 1965), p. 29.

41 Laura King, ‘Hiding in the Pub to Cutting the Cord? Men's Presence at Childbirth in Britain, *c*.1940s–2000s’, *Social History of Medicine* 30 (2017), pp. 389–407.

42 Debbie Singh and Mary Newburn, *Becoming a Father: Men's Access to Information and Support about Pregnancy, Birth, and Life with a New Baby* (London: National Childbirth Trust, 2000), p. 49. It is difficult to know exact proportions of attendance, as records often do not differentiate between presence at labour or delivery, and the presence of fathers or other relatives/friends.

43 King, ‘Hiding in the Pub’.

44 For example, Jackson, *Fatherhood*; Lewis, *Becoming a Father*; Newson and Newson, *Patterns of Infant Care*, pp. 133–47; Elizabeth Roberts, *Women and Families: An Oral History, 1940–1970* (Oxford: Wiley‐Blackwell, 1995), esp. pp. 154–7; Lynne Segal, *Slow Motion: Changing Masculinities, Changing Men* (Basingstoke: Palgrave Macmillan, 2007), pp. 23–49.

45 King, ‘Now You See’, p. 600.

46 Colin Bell, Lorna McKee and Karen Priestley, *Fathers, Childbirth and Work: A Report of a Study* (London: Equal Opportunities Commission, 1983), p. 6. Nearly half took one to two weeks off, and the authors found that 91.7 per cent of fathers asked favoured a formal paternity leave scheme, p. 65.

47
*Daily Mirror*, 24 May 1982, p. 9. This ranged from one day for British Rail workers to six weeks for fathers who worked for *Time Out* magazine.

48 King, *Family Men*, pp. 52–7, 185–6; John Tosh, *A Man's Place: Masculinity and the Middle‐Class Home in Victorian England* (New Haven: Yale University Press, 1999), pp. 87–90; Williamson, ‘He was Good’, p. 98.

49 Davis, *Modern Motherhood*, pp. 198–9.

50 Jeffrey Weeks, *Sex Politics and Society* (1981; 3rd edn London: Longman, 2012), p. 360.

51 Irene Bruegel and Diane Perrons, ‘Deregulation and Women's Employment: The Diverse Experiences of Women in Britain’, *Feminist Economics* 4 (1998), pp. 103–25.

52 Lynn Abrams, *The Making of Modern Woman: Europe, 1789–1918* (London: Routledge, 2002), p. 13.

53 Elizabeth Roberts, *A Woman's Place: An Oral History of Working‐Class Women, 1880–1940* (London: Blackwell, 1984), p. 110.

54 Roberts, *Women and Families*, p. 90–2.

55 Diana Gittins, ‘A Woman's Place: An Oral History of Working‐Class Women 1890–1940/Women and Families: An Oral History, 1940–1970’, *Sociology* 29 (1995), pp. 768–70, here p. 769.

56 For an exploration of this in an earlier period, see King, *Family Men*, ch. 3, pp. 51–88.

57 For example, Jane Lewis has shown the similarities between the United Kingdom and the Netherlands in terms of patterns of work and care, and has compared women's work in Britain and the United States. Jane Lewis, ‘The Decline of the Male Breadwinner Model: Implications for Work and Care’, *Social Politics* 8 (2001), pp. 152–169; Margaret Walsh and Chris Wrigley, ‘Womanpower: The Transformation of the Labour Force in the UK and the USA since 1945’, *ReFRESH* 31 (2001), pp. 1–4.

58 Sian Pooley and Kaveri Qureshi (eds), *Parenthood Between Generations: Transforming Reproductive Cultures* (New York: Berghahn Books, 2016).

59 Jane Lewis, ‘Gender and Welfare State Change’, *European Societies* 4 (2002), pp. 331–57, here, p. 332.

## Appendix

Date and region of interviewee's birth; date and region of biological children's birth (including children who died in childbirth or infancy); level of education. (Exact places of birth have been removed to preserve anonymity).

English Regions:

NE: North East

NW: North West

WM: West Midlands

EM: East Midlands

SW: South West

EE: East of England

SE: London and South East

YH: Yorkshire and the Humber

Level of Education:

Secondary: Left education aged 16 or under.

Further: post‐16 qualification

Higher: university level qualification

Arranged in order of date of birth of first child:

1945–1954

Glenda – b.1927 SE. Three children: 1952 SE; 1954 SE; 1958 SE. Education: Further.

Harry – b.1924 YH. Two children: 1948 YH; 1952 YH. Education: Secondary.

Tania – b.1931 SE. Four children: 1952 SE; 1953 SE; 1954 SE; 1962 SE. Education: Secondary.

1955–1964

Alf – b.1933 SE. Two children: 1961 YH; 1964 YH. Education: Secondary.

Brian – b.1928 WM. Two children: 1964 YH; 1967 YH. Education: Higher.

Camilla – b.1937 YH. Three children: 1961 SE; 1963 WM; 1965 WM. Education: Higher.

Claire – b.1933 SE. Six children: 1960 SE; 1961 SE; 1963 SE; 1965 SE; 1969 SE; 1972 SE. Education: Higher.

Diana – b.1931 NW. Three children: 1958, SE; 1959 SE; 1964 Yemen. Education: Secondary.

Eve – b.1927 NW. Two children: 1957 NW; 1960 NW. Education: Secondary.

Fred – b.1928 YH. One child: 1961 YH. Education: Higher.

Harold – b.1931 EM. Two children: 1962 YH; 1964 YH. Education: Secondary.

Hayley – b.1926 EE. Three children: 1956 EE; 1958 YH; 1959 SE. Education: Higher.

Marjorie – b.1931 SE. Four children: 1959 SE; 1961 SE; 1964 SE; 1966 SE. Education: Higher.

Penny – b.1937 NW. Three children: 1955 EE; 1957 SE; 1964 SE. Education: Secondary.

Stephanie – b.1938 SE. Four children: 1962 Germany; 1963 SE; 1965 SE; 1972 SE. Education: Further.

Yvonne – b.1940 SE. Four children: 1959 SE; 1961 SE; 1963 SE; 1977 SE. Education: Further.

1965–1974

Amanda – b.1940 SW. Four children: 1965 SW; 1966 SW; 1968 WM; 1973 SE. Education: Further.

Barry – b.1935 SE. Two children: 1972 YH; 1976 YH. Education: Higher.

Ben – b.1943 SW. Three children: 1970 SE; 1972 SE; 1983 YH. Education: Higher.

Fiona – b.1941 WM. Three children: 1966 SE; 1968 SE; 1970 SE. Education: Higher.

George – b.1943 SW. Two children: 1973 YH; 1975 YH. Education: Higher.

Henry – b.1942 WM. Four children: 1966 EM; 1968 YH; 1970 YH; 1975 YH. Education: Higher.

Hilda – b.1942 NE. Two children: 1967 SE; 1970 SE. Education: Secondary.

Karen – b.1945 SE. Four children: 1967 SE; 1970 SE; 1984 WM; 1985 WM. Education: Higher.

Kelly – b.1935 India. Two children: 1968 SE; 1970 NW. Education: Higher.

Liz – b.1946 NW. Three children: 1973 Germany; 1975 Northern Ireland; 1980 WM. Education: Further.

Lynne – b.1946 Wales. One child: 1973 Canada. Education: Further.

Margaret – b.1944 EE. Two children: 1969 SE; 1970 SE. Education: Further.

Mike – b.1942 YH. Two children: 1971 NE; 1974 NE. Education: Further.

Nick – b.1942 Zambia. Three children: 1972 EE; 1973 Malawi; 1979 YH. Education: Higher.

1975–1984

Alex – b.1950 YH. Four children: 1979 YH; 1982 YH; 1983 YH; 1986 YH. Higher.

Donald –b.1941 SE. One child: 1983 YH. Education: Higher.

Edward – b.1940 YH. One child: 1976 YH. Education: Higher.

Frank – b.1952 NW. Three children: NW 1975; YH 1983; YH 1984. Education: Higher.

Gina – b.1951 SE. Two children: 1978 WM; 1980 SE. Education: Higher.

Hermione – b.1948 WM. Three children: 1976 SE; 1979 SE; 1984 SE. Education: Further.

Malcolm – b.1949 NW. Two children: 1975 YH; 1979 YH. Education: Higher.

Mick – b.1947 SE. One child: 1977 YH. Education: Higher.

Pam – b.1947 Wales. Two children: 1977 SE; 1983 SE. Education: Secondary.

Reg – b.1946 SE. Three children: 1979 YH; 1986 YH; 1988 YH. Education: Higher.

1985–1990

Bev – b.1954 SE. Two children: 1987 SE; 1990 SE. Education: Higher.

Daniel – b.1961 United States. Three children: 1985 YH; 1987 YH (twins). Education: Higher.

James – b.1962 WM. Two children: 1986 WM; 1989 YH. Education: Higher.

Simon – b.1963 NW. Three children: 1987 NW; 1989 NW; 1990 NW. Education: Further.

